# Can Manganese Dioxide Microspheres be Used as Intermediaries to Alleviate Intervertebral Disc Degeneration With Strengthening Drugs?

**DOI:** 10.3389/fbioe.2022.866290

**Published:** 2022-04-01

**Authors:** Wentao Zhang, Ming Yang, Tianze Sun, Jing Zhang, Yantao Zhao, Jingmin Li, Zhonghai Li

**Affiliations:** ^1^ Department of Orthopedics, First Affiliated Hospital of Dalian Medical University, Dalian, China; ^2^ Key Laboratory of Molecular Mechanism for Repair and Remodeling of Orthopedic Diseases, Dalian, China; ^3^ Department of Orthopedics, Fourth Medical Center of PLA General Hospital, Beijing, China; ^4^ Beijing Engineering Research Center of Orthopedics Implants, Beijing, China; ^5^ Key Laboratory for Micro/Nano Technology and System of Liaoning Province, Dalian University of Technology, Dalian, China

**Keywords:** degenerative disc disease (DDD), intervertebral disc (IVD), manganese dioxide (MnO_2_) nanoparticles, cytokines, therapeutic protein injection

## Abstract

Degenerative disc disease (DDD) is a pathological condition associated with intervertebral discs (IVDs) that causes chronic back pain. IVD degeneration has become a significant issue in contemporary society. To date, numerous biological therapies have been applied to alleviate the progression of DDD, among which therapeutic protein injection is the most direct and convenient. However, there are some limitations to applying direct protein injection therapy, the most significant being that the efficacy of this method has a short duration, which is a major factor in its effectiveness and the resulting patient satisfaction. How do we solve this problem? Or how can the effectiveness of the treatment be enhanced? It has been proved that manganese dioxide (MnO_2_) microspheres, widely used in environmental science, not only regulate the expression of cell genes and cytokines in the microenvironment, but also have the ability to release drugs slowly. We propose that direct injection of protein encapsulated in hollow MnO_2_ (h-MnO_2_) microspheres could solve the problem of rapid drug release. In addition, the use of a MnO_2_ and protein injection in the treatment of DDD may have a synergistic effect, which would be highly significant for the degradation of pro-inflammatory factors in the DDD microenvironment. Therefore, the combination of MnO_2_ and protein may provide a new therapeutic approach to alleviate the progression of DDD.

## Introduction

The nucleus pulposus (NP) in the normal intervertebral disc (IVD) is surrounded by the peripheral anulus fibrosus (AF), which has no blood passing through it, and its mass exchange is determined by the concentration gradients of different molecules around it—such as glucose, oxygen, and other branches—through the peripheral cartilage endplate and AF. Owing to the nature of the NP cells farthest from the blood supply, which determines the hypoxic environment in the IVD, NP cells prefer anaerobic metabolism. Therefore, the NP microenvironment has a higher lactate concentration and a lower PH than other IVD sites, and this environment can have a negative effect on cell metabolism and function (Benneker et al., 20042005). When the lumbar disc is degenerative, the NP can break through the posterior margin of the AF and contact or enter the epidural space under the action of external force. The NP itself has antigenicity, coupled with the inflammatory response caused by structural changes, which can stimulate the immune system, resulting in discomfort and pain. The pain can be quite intense, placing significant physical and mental burdens on the patient. In addition, 70–90% of the composition of the NP is water, and IVD tissue itself is hyperosmolar; therefore, the protruding NP absorbs water and expands after contact with the epidural spinal fluid, resulting in further enlargement of the protrusion, and causing the signal strength of images to change from high to low (Manchikanti et al., 2015). Many studies have shown that abnormal cell apoptosis plays an important role in degenerative disc disease (DDD); however, neuropathic pain and inflammatory response may also have a close relationship with DDD. The presence of multiple inflammatory cell responses in the herniated IVD tissue suggests that inflammatory response is involved in the occurrence of lumbar disc herniation and may be the most important mechanism (Zhang et al., 2017; Zhao et al., 2017). Molecular immunology and molecular biology studies have shown that inflammatory mediators can cause lumbar IVD degeneration, in other words, the degenerative lumbar IVD tissue can release interleukin (IL), tumor necrosis factor-α (TNF-α), and other inflammatory mediators, which stimulate the accumulation and activation of inflammatory cells and release numerous inflammatory transmitters, inducing osteoarticular neuropathic pain and participating in the pathogenesis and progression of lumbar disc herniation (Lu et al., 2020; Qi et al., 2020; Jiang et al., 2021). It has been reported that significantly higher levels of IL-1, IL-6, and TNF were detected in serological analysis in DDD models than in control groups. IL-1β can regulate the activity of matrix metalloproteinases (MMPs) and inhibit the synthesis of proteoglycan by the cell matrix, thus participating in the process of DDD. IL-6, a typical inflammatory mediator, works by interfering with degrading enzymes, which can change the level, structure, type, and function of biomacromolecules such as elastin, proteoglycan, and collagen in the IVD matrix, resulting in the AF showing weakened protection and promoting the protrusion of NP from the weak AF. TNF-α, a strong inflammatory cytokine, can up-regulate the gene expression and activity of MMPs, and stimulate IL-6, IL-8, and other related cytokines. Moreover, it can promote cell migration, affect the permeability of endothelial cells, block the synthesis of collagen and proteoglycan, and induce inflammation.

Currently, there are several biological approaches for the treatment of DDD, such as protein injection therapy, in which the injected protein solution mediates cell growth and/or anabolic reactions in the IVD (Thompson et al., 1991; Moriguchi et al., 2016); gene therapy, which induces targeted gene expression in the IVD (Nishida et al., 2008); cell-based therapy, which reverses the cascade of degeneration using stem cells and other allogeneic cells (Clarke et al., 2014); and tissue engineering therapy, which introduces a functional replacement scaffold into damaged IVD tissue (Xin et al., 2013; Martin et al., 2017). The degeneration of IVDs can be delayed by appropriate interventions. As the mechanisms of degeneration are complex and diverse, intervention methods range from simple to more sophisticated, with varying effects. With advances in research into the degeneration of the IVD, new modes of treatment are emerging and being verified. The current treatment methods need to be optimized to regulate IVD cells in a more targeted manner.

Manganese dioxide (MnO_2_) is a common mineral with many unique chemical and physical properties. Owing to their low toxicity, strong adsorption, and good biocompatibility, MnO_2_ microspheres can carry a variety of drugs and cytokines, then degrade into manganese ions under weakly acidic conditions to slowly release the encapsulated cargo and achieve a therapeutic effect. Many studies have shown that MnO_2_ has a positive effect on microenvironment regulation. Hydrogen peroxide (H_2_O_2_) can stimulate the body and over activate pro-inflammatory immune cells. In Rajendrakumar’s study, they prepared a mannosylated-polymericalbumin-manganese dioxide (mSPAM) nano-assembly, as a peroxide scavenger to catalyze the decomposition of H_2_O_2_. The results suggested that the mSPAM nano-assembly inhibited the activation of NF-Kβ mediated by HIF-1α, while reducing the H_2_O_2_ concentration, thus achieving an anti-inflammatory effect and inhibiting local and systemic inflammatory manifestations and the development of neuroinflammation (Rajendrakumar et al., 2018). In addition, the level of glutathione (GSH) —a factor that can catalyze the decomposition of MnO_2_—is always higher in cancer cells than in normal cells (Gamcsik et al., 2012), in general, since hollow MnO_2_ (h-MnO_2_) can be degraded by intracellular GSH to form Mn^2+^ with excellent Fenton-like activity to generate highly reactive hydroxide (He et al., 2021; Ou et al., 2021). Under the cancer cell environment, the released Mn^2+^ exhibited strong chemodynamic effect through Fenton-like reaction, further over-expressed GSH is consumed, the depletion of GSH further improved the chemodynamic therapy efficiency (Yang et al., 2014; Gao et al., 2022; Ma et al., 2022). Therefore, therefore, h-MnO_2_ microspheres may have a faster degradation rate in cancer cells, allowing more efficient and accurate release of drugs in target cancer cells. Degradation of h-MnO_2_ occurs very rapidly, however, one of the major advantages of the system is that the degradation rate can be manipulated using the particle geometry or the wall thickness, thus allowing control of the drug release (Li et al., 2020; Greene et al., 2021).

## Hypothesis

During the process of IVD degeneration, the types of cytokine and the expression of various genes in the microenvironment alter. Currently, MnO_2_ microspheres are predominantly used in environmental studies; however, it has gradually emerged that MnO_2_ has marked effects in biology, particularly in anticancer applications. Studies have shown that MnO_2_ microspheres can regulate the expression of genes and the number of cytokines in specific microenvironments. The expression of cells during IVD degeneration coincides with the regulation of cytokines in the environment by MnO_2_ microspheres; therefore, it is assumed that direct injection of protein encapsulated in h-MnO_2_ microspheres can alleviate the progression of IVD degeneration.

## The Process of IVD Degeneration

**FIGURE 1:**
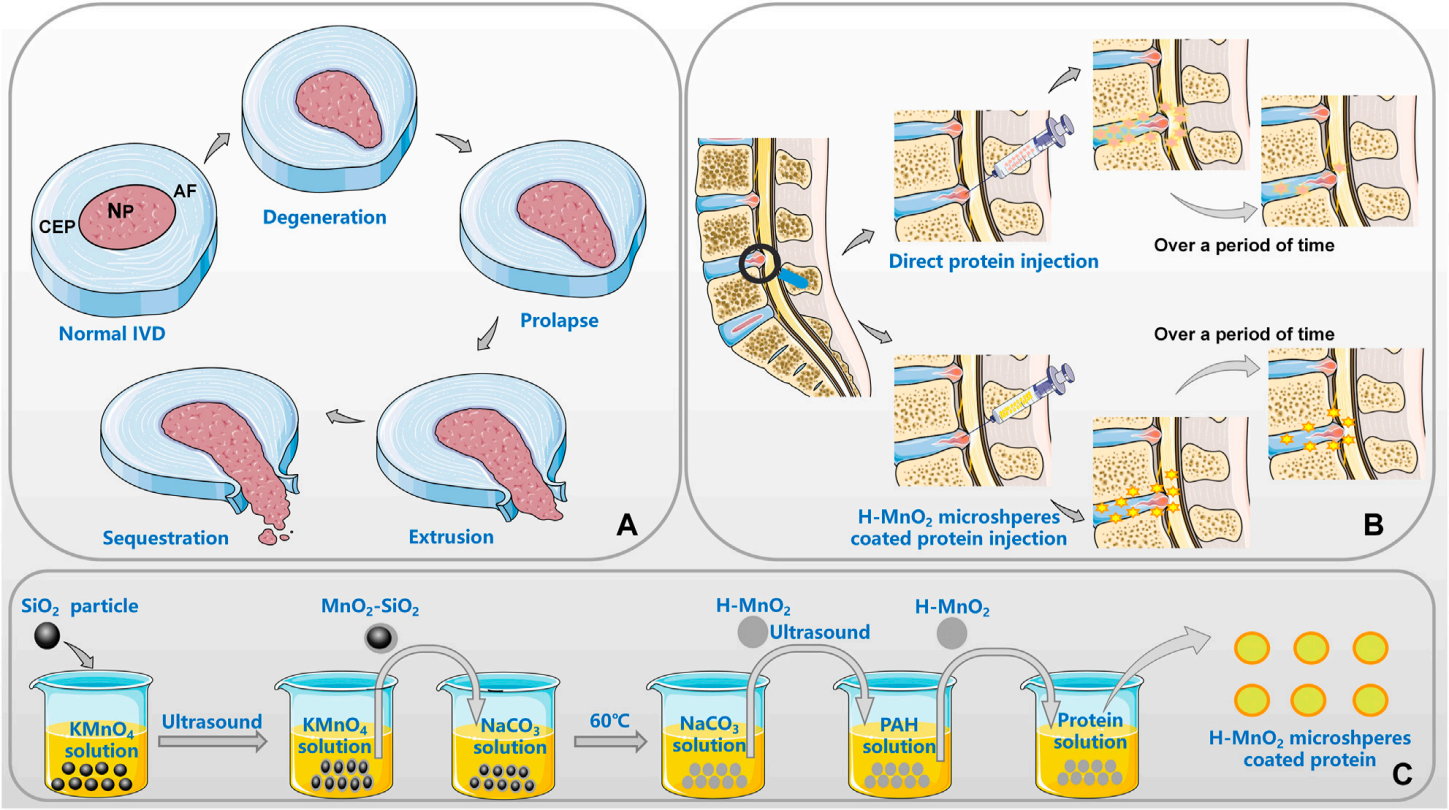
Application of MnO_2_ microspheres in DDD [**(A)**. the pathological progress of IVD; **(B)**. the advantages of MnO_2_ as a carrier; **(C)**. the general preparation process of the h-MnO_2_ protein delivery].

DDD encompasses a series of diseases that primarily manifest as chronic lower back pain, which is closely related to IVD changes. There are different mechanisms that lead to DDD, such as vertical genetic inheritance, mechanical damage to an acquired development, and unavoidable physical exposure. Although each stage is different, these pathways lead to a common outcome that promotes DDD: an imbalance in the synthesis and catabolism of the extracellular matrix (ECM), in favor of catabolism. The degeneration of IVDs is not the result of a single simple factor, but that of the interaction of the genes, environment, and physics, among other factors, making it a complex process. DDD typically involves a chronic process of change, including a progressive decrease in the supply of nutrition to the IVD and gradual changes in the composition of the ECM (Figure 1). The former has been shown to have a negative effect on the maintenance of the ECM, resulting in a further decrease in oxygen concentration and a lower pH in the IVD. The change in the latter leads to reduced tissue strength, which affects the metabolism and function of cells (Nachemson et al., 1970). At the same time, nutritional deficiency weakens the response of IVD to stimulation. However, genetic factors may play a more important role in DDD than mechanical injury and malnutrition (Kalichman and Hunter, 2008; Livshits et al., 2011). Genes leading to DDD can be divided into different types according to their functions (Mayer et al., 2013). For example, genes which affect the structure of IVD: Aggrecan (ACAN), COL1, COL9, COL11, FN, HAPLN1, thrombospondin, cartilage intermediate layer protein (CILP), and asporin (ASPN); catabolic genes: MMP1, MMP2, MMP3, PARK2, and PSMB9; and anticatabolic tissue inhibitors of metalloproteinases (TIMPs). The polymorphism and type of genes affect the delicate balance between synthesis and catabolism in the IVD, leading to IVD degeneration. Of course, factors that increase the inflammatory cascade can also upset the biochemical balance of the internal environment, thus accelerating the degeneration of the IVD. Polymorphisms within IL-1, IL-6, and COX-2 have been confirmed to be associated with DDD.

The dynamic balance between anabolism and catabolism is fundamental to the normal physiological effects of the IVD. This balance is regulated by the products of anabolism (TGF, IGF, etc.) and catabolism (MMPs, ADAMTS, HRTA1, etc.), and inhibitors of them. Excessive catabolism in the ECM of IVD leads to the increase of degradation products and triggers the overexpression of inflammatory mediators, thus aggravating the increase of degradation products and creating a vicious cycle (Sakai and Grad, 2015). Catabolic enzymes are expressed differently in different states of IVD degeneration, and these differences can be regulated by immobilization or mechanical overloading. For example, in normal IVDs, MMP1 and ADAMTS4, which play an important role in maintaining homeostasis *in vivo*, show low expression, while MMP3 and MMP13 are almost unexpressed. In degenerative IVDs, the expression of MMP1, MMP3, MMP13, and ADAMTS4 is significantly increased, and the degree of increase is proportional to the disease.

In addition, there are many factors that can affect the homeostasis of the IVD internal environment. For example, cell senescence also disrupts the balance between ECM synthesis and catabolism (Gruber et al., 2009), and oxidative stress in the IVD microenvironment can accelerate the degeneration process. Changes in the reactive oxygen species (ROS) concentration are closely related to the degree of DDD, while, as the main source of ROS during IVD degeneration, the proliferation state of NP cells and activation of the senescent signal pathways to induce cell cycle arrest of NP cells, can affect the ROS content. The mechanical pressure of the IVD also increases the concentration of local ROS. In summary, various factors can lead to IVD degeneration through different pathways (Martin et al., 2004).

IVD protein injection therapy, where protein solution is directly injected into the IVD to alter the metabolism and development of cells to delay or reverse the occurrence of degeneration, has been widely used in DDD. For example, direct injection of IGF-1 into the IVD can prevent cell senescence caused by oxidative damage; direct injection of growth factors can limit endplate calcification; and intra-IVD injection of OP-1 can increase proteoglycan secretion and increase IVD height (Bibby and Urban, 2004; Imai et al., 2007; Gruber et al., 2008). However, there are some limitations to applying direct protein injection therapy, the most significant being that the efficacy of this method has a short duration, which is a major factor in its effectiveness and the resulting patient satisfaction. The efficacy of direct protein injection therapy can be effectively enhanced by some means to prolong the time of drug action. At present, the microsphere technology is developing rapidly, and a large number of studies have shown that the application of microsphere technology to drug encapsulation can prolong the action time of drugs *in vivo*.

## Anti-Inflammatory Effect OF MnO_2_ Microspheres *In Vivo*

Motivated by the physicochemical properties of MnO_2_, Shreedevi’s group developed and characterized ROS-scavenging MnO_2_ microspheres (Kumar et al., 2019). The microspheres play a cartilage protection role in cartilage explants by reducing nitric oxide release and maintaining the glycosaminoglycan content, in other words, the particles counter oxidative stress by reducing the expression of genes associated with aggressive cytokines. ROS-scavenging microspheres made of MnO_2_ have natural physicochemical properties in articular cartilage, allowing them to permeate freely in the cartilage and maximizing their retention time in the joint. H_2_O_2_, a free radical derived from O_2_^−^, is one of the main active oxidizing species produced by chondrocytes (Broughton et al., 1947). MnO_2_ catalyzes the decomposition of H_2_O_2_ into oxygen and water, which can relieve the oxidative stress reaction and provide an oxygen equivalent for cells. As we all know, the destruction of hypoxic microenvironment of NP cells plays a critical role in the pathogenesis of IVD degeneration. In NP cells, the treatment of high oxygen tension (HOT) leads to upregulation of integrin alpha 6 (ITG-α6) expression, which can be alleviated by blocking the PI3K/AKT signaling pathway. ITG-α6 can protect NP cells against HOT-induced apoptosis and oxidative stress and protect NP cells from HOT-inhibited ECM protein synthesis. Upregulation of ITG-α6 expression by HOT helps maintain NP tissue homeostasis through the interaction with HIF-1α (Kim et al., 2021; Zheng et al., 2021). It was found that the expression levels of ITG-α6 were increased in NP tissue of IVD degeneration patients and IVD degeneration rat model with mild degeneration, which could produce the above-mentioned effects to protect NP cells from hyperoxia (Xu et al., 2021). In addition, MnO_2_ particles can protect Langerhans islet cells (Tootoonchi et al., 2017). Most importantly, MnO_2_ microspheres coated with drug can be implanted in the body to achieve a slow-release effect and thus address the issue of rapid drug release. To enhance the colloidal stability of MnO_2_ particles in biological fluids, MnO_2_ microspheres can be coupled with polyethylene glycol (PEG) to form PEG-MnO_2_ particles, thus enhancing the biocompatibility of MnO_2_ particles with the human body.

IL-1β plays a destructive role in osteoarthritis and enhances catabolism. IL-1β stimulates the production of catabolic enzymes like MMPs, thereby degrading the ECM and leading to the release of GAGs (Daheshia and Yao, 2008). In addition, it stimulates the production of another destructive mediator—nitric oxide synthase—which inhibits the production of proteoglycans and collagen components, raises the expression of NO, and induces chondrocyte cell death (Abramson, 2008). Experiments show that a certain concentration of PEG-MnO_2_ microspheres can inhibit GAGs and NO release in cartilage that is stimulated by IL-1β. The catabolic enzymes MMP1 and MMP13 are effective matrix degrading enzymes and major catabolic factors in osteoarthritis that can lyse COL II in cartilage, leading to collagen degradation and GAGs loss (Rose and Kooyman, 2016). When chondrocytes were attacked by cytokines, the expression of MMP1 and MMP13 was upregulated tens or even hundreds of times. However, their expression was maintained at a normal baseline after treatment with PEG-MnO_2_ microspheres. ADAMTS5 is a key enzyme in proteoglycan degradation. In cell experiments, IL-1β stimulated the expression of ADAMTS5 disintegrin and up-regulated metalloproteinase. However, in the PEG-MnO_2_ microspheres with IL-1β group, the amounts of all three substances remained at baseline level without significant change. The expression of thioredoxin-1 (TXN1) was upregulated in both osteoarthritis and rheumatoid arthritis tissues (Abramson, 2008), and TXN1 is widely involved in redox reactions in cells. The expression of TXN1 was enhanced in all chondrocytes co-cultured with IL-1β, while in the group co-cultured with PEG-MnO_2_ microspheres, the expression of TXN1 was not notably different from normal. In addition, PEG-MnO_2_ microspheres have been shown to down-regulate secretion of the pro-inflammatory factor TNF-α in a dose-dependent manner (Kumar et al., 2019). Furthermore, nano-sized MnO_2_ particles not only play a role in tumor therapy (Liang et al., 2018; Zeng et al., 2019) and regulating cytokine expression in the microenvironment, but also have newly demonstrated uses in medical imaging and radiotherapy (Sun et al., 2016). Studies have shown that Mn^2+^ ions are generated when MnO_2_ nanostructures are decomposed in the presence of H^+^ or GSH, and the generated Mn^2+^ ions significantly improve the imaging contrast of T1-magnetic resonance (MR) (Platas-Iglesias et al., 2016). In addition, drug-loaded h-PEG-MnO_2_ was incubated in different pH buffer solutions for 6 h for MR imaging. It was found that the signal of microspheres in neutral buffer solution was weak, while the drug-loaded h-PEG-MnO_2_ sample had an acid concentration dependent brightening effect.

**FIGURE 2:**
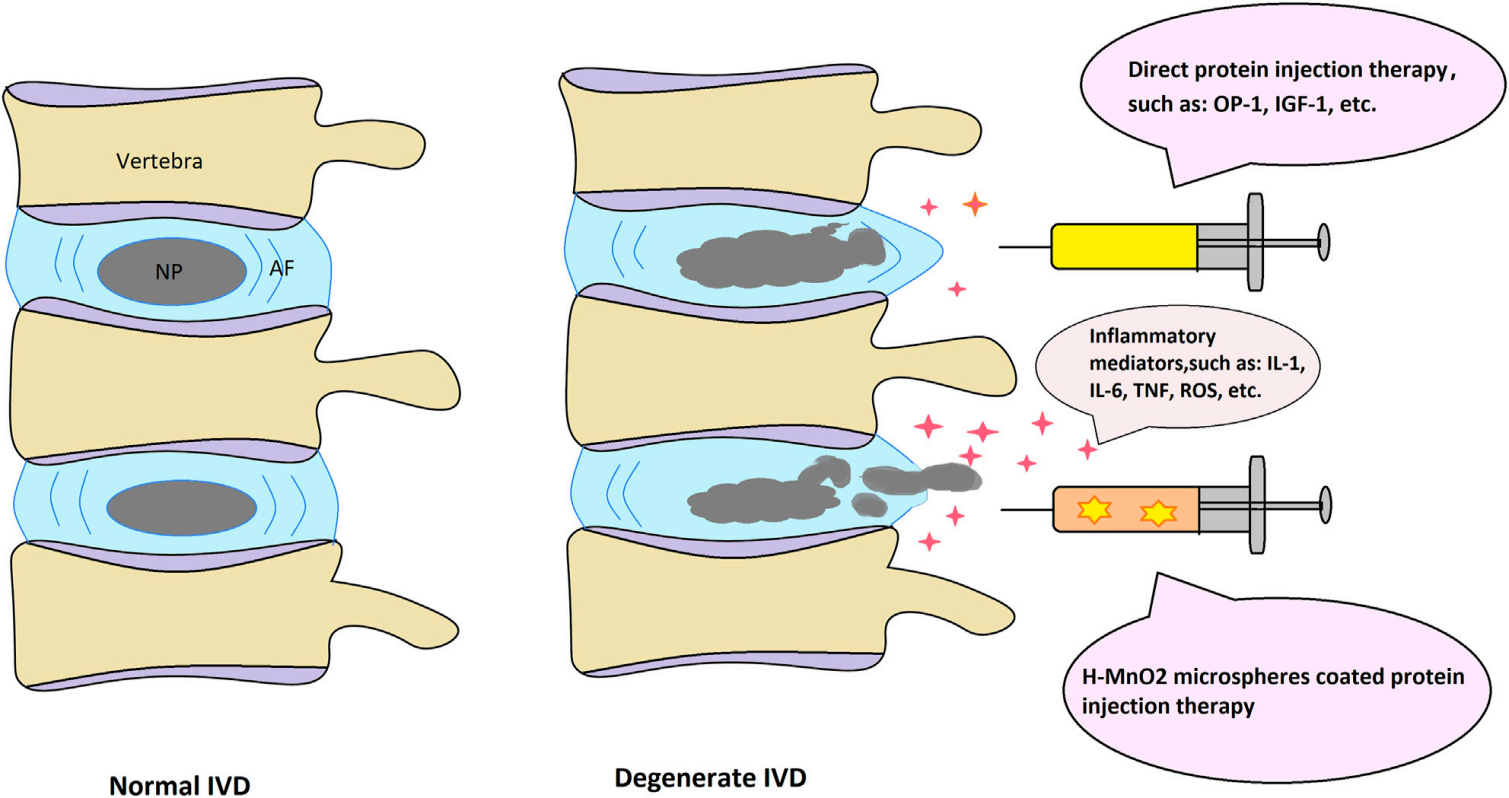
Schematic depiction of the normal and degenerate IVD structure with possible protein injection therapeutic approaches.

Nano-sized MnO_2_ particles have attracted significant attention in the field of anticancer on account of their favorable properties. Treatment with h-MnO_2_ microspheres led to a reduction in cell numbers. The number of cancer cells showed a greater decrease than that of healthy cells; however, it must be noted that MnO_2_ microspheres also damage normal cells (Figure 2).

In conclusion, MnO_2_ microspheres can relieve the oxidative stress reaction in organisms, provide an oxygen equivalent for cells, improve the low oxygen concentration and low PH state in microenvironment, and thus restore the tissue strength and the process of cell metabolism. At the same time, MnO_2_ particles can also regulate the production of cytokines from the level of regulating genes to reduce the degree of inflammation, in this process, MnO_2_ is gradually decomposed into Mn^2+^, which are excreted with body fluids, so as to restore the internal environment of the body to the optimal state. MnO_2_ can change the living environment of anaerobic nucleus pulposus cells from the perspective of gene expression, and realize the repair of IVD. Most importantly, MnO_2_ microspheres can achieve the effect of sustained release of encapsulated drugs, and cooperate with the drug mechanism to resist the negative effects caused by various pro-inflammatory factors (Figure 1).

## Conclusion

The release of pro-inflammatory cytokines such as IL-1 in the IVD leads to the accumulation of ROS, which manifests as increased production of hydroxylated radicals, peroxides, and NO. In addition, the increase of ROS down-regulates the expression of antioxidants such as superoxide dismutase, catalase, and glutathione peroxidase. This series of changes leads to enhanced catabolism of IVD tissues, reduced matrix synthesis and enhanced ECM degradation, local inflammatory reaction, and gradual senescence or necrosis of cells, and further aggravates the disease and symptoms. At the same time, a variety of other cytokines and gene expressions in the microenvironment change during the process of IVD degeneration. Currently, MnO_2_ microspheres are primarily used in environmental studies; however, reports are gradually beginning to show that MnO_2_ has marked effects in biology, particularly as an anticancer agent. It has been shown that MnO_2_ microspheres can regulate the expression of genes and the production of cytokines in a specific environment and have the ability to slow drug release. In summary, the expression of cells during IVD degradation overlaps with the MnO_2_ regulation of cytokines in the local environment. It is now hypothesized that direct injections of protein encapsulated in h-MnO_2_ microspheres may be superior to direct injections of protein for the alleviation of IVD progression.
